# How Can Digital Health Technologies Contribute to Sustainable Attainment of Universal Health Coverage in Africa? A Perspective

**DOI:** 10.3389/fpubh.2019.00341

**Published:** 2019-11-15

**Authors:** Olushayo Olu, Derrick Muneene, Juliet Evelyn Bataringaya, Marie-Rosette Nahimana, Housseynou Ba, Yves Turgeon, Humphrey Cyprian Karamagi, Delanyo Dovlo

**Affiliations:** ^1^WHO Country Office, Juba, South Sudan; ^2^WHO Regional Office for Africa, Brazzaville, Republic of Congo; ^3^WHO Country Office, Kigali, Rwanda; ^4^International Health System Strengthening Expert, Accra, Ghana

**Keywords:** digital health, e-Health, sustainable development goals, universal health coverage, resilient health systems, Africa

## Abstract

**Background:** Innovative strategies such as digital health are needed to ensure attainment of the ambitious universal health coverage in Africa. However, their successful deployment on a wider scale faces several challenges on the continent. This article reviews the key benefits and challenges associated with the application of digital health for universal health coverage and propose a conceptual framework for its wide scale deployment in Africa.

**Discussion:** Digital health has several benefits. These include; improving access to health care services especially for those in hard-to-reach areas, improvements in safety and quality of healthcare services and products, improved knowledge and access of health workers and communities to health information; cost savings and efficiencies in health services delivery; and improvements in access to the social, economic and environmental determinants of health, all of which could contribute to the attainment of universal health coverage. However, digital health deployment in Africa is constrained by challenges such as poor coordination of mushrooming pilot projects, weak health systems, lack of awareness and knowledge about digital health, poor infrastructure such as unstable power supply, poor internet connectivity and lack of interoperability of the numerous digital health systems. Contribution of digital health to attainment of universal health coverage requires the presence of elements such as resilient health system, communities and access to the social and economic determinants of health.

**Conclusion:** Further evidence and a conceptual framework are needed for successful and sustainable deployment of digital health for universal health coverage in Africa.

## Background

The sustainable development goals (SDGs) are ambitious, universal, and applicable to all countries. They encompass the most contemporary development issues and are grounded on the principles of equity (leave no one behind), human rights, accountability and sustainability. The same principles underpin global work in health and are central to the current drive for universal health coverage (UHC), the umbrella target for the third SDG (1). UHC means that all individuals and communities receive the health services they need, at good quality and without suffering financial hardship (1, 2).WHO recognizes that this is a priority to accelerate the attainment of SDG 3 (3, 4). There are however, many impediments to attaining UHC in Africa^1^, including but not limited to the high operational and financial costs needed to expand access to many areas where there is no current access, while sustaining an acceptable level of quality of services (5). Other impediments include pervasively weak health systems, inadequate physical access to several communities as a result of difficult terrain and insecurity, poor infrastructure, transport and sociocultural barriers. Current service delivery approaches may not deliver on universal health coverage because of these impediments. Innovative approaches to deliver services which can ensure universal coverage with essential services, in current social and economic environments are therefore needed (6).

In recent times, Digital Health (DH) has gained a lot of traction globally as an engine for innovation to address these challenges and leapfrog attainment of the SDGs and UHC (5, 7–9). Subsequently, several DH initiatives and pilot projects have been successfully implemented in many countries in Africa. The lessons learnt from these DH initiatives shows that they are a means to an end and not an end in themselves and should thus be context specific and results driven (10). Furthermore, because DH sits at the nexus of two key sectors namely health and information and communication technologies (ICT), there are associated knowledge, coordination, and communication gaps about the topic in both sectors.

In this article, we analyse the key benefits and challenges associated with the use of DH for health system strengthening and contribution toward the attainment of UHC in Africa. We examine the key prerequisites and propose a conceptual framework for the effective scale up of DH and their use as tools to achieve UHC with the aim of raising awareness amongst African public health and ICT stakeholders, decision makers, public health managers and workers. Last, we propose future directions for scaling up the use of DH for UHC on the continent.

## Digital Health: Evolution, Applications, Benefits, and Challenges in Africa

DH refers to the use of ICT for health (11, 12). In the context of this paper, this term is used synonymously with a similar term, eHealth. DH could be applied to several areas of medicine and public health including patient and public health data management (electronic health records), provision of remote health care services (telemedicine/telehealth), health information and services through mobile telephone technology (mHealth), health knowledge management and distant learning for health workers (eLearning). Other applications include connection of medical devices (internet of things), improved planning, organization, and management of health services particularly at the sub-national level, and more recently to the management of large public health data (13). DH could also be used to improve personal health through the use of wearable devices which monitor, analyse, and transmit vital signs to personal or central repositories. Programmatically, DH has been successfully applied to the prevention of non-communicable diseases including cancers (14), maternal and child health (15), immunization (16), HIV/AIDS management (17), essential medicines and medical products supply chain management (18) among others. These various applications of DH have shown promising results and potential for scale up, a feature not so prevalent in many African countries.

Use of digital technologies for health has potential benefits including improving access to health care services especially for those in hard-to-reach areas, improvements in safety and quality of healthcare services and products, improved knowledge and access to health information for health workers and communities leading to better productivity of the health workforce, and increased uptake of health services (19–21). Furthermore, digital technologies can improve efficiency and reduce the cost of health services delivery (21), facilitate rapid transmission of public health information in real time for timely decision making and enhance the capacity for monitoring the performance of programmes and the health system as a whole. Other potential benefits include detecting and addressing sociocultural, physical, and financial barriers to equitable access to health and digitalization of health insurance schemes which could make them more efficient. DH thus has the potential to expand good and affordable health services to the last mile, a prerequisite for UHC. However, these benefits accrue and are realized when DH are aligned with national heath priorities, development goals, and more also when they meet citizen needs (22). In addition, these benefits would deliver much more value when DH services are offered at scale, is context specific and cost-effective (19).

Remarkable achievements in the sphere of ICT have created an enabling environment for widespread deployment of DH in Africa. For instance, it is estimated that about 95% of the world's population now live within areas which are well served by mobile networks and that there are more than seven billion mobile subscriptions globally (an average of one per person) (23). However, at 74% (24) and 21.8% (25) respectively, mobile phone and internet penetration in Africa is lower than the global average but sufficient for scale up of DH on the continent.

The leading DH solutions on the continent include mHealth, eLearning, and telehealth. Social media and electronic health records are also gaining ground (26). New intergovernmental arrangements between Ministries of Health and ICT have also been initiated. A number of countries in the region have reported implementation of digital medical devices, which are key elements of digital clinics and the creation of digital patients (27). The digital clinics themselves are generally characterized by use of medical devices to support remote diagnostic processes and procedures, thereby reducing the gap brought about by the lack of expert health professionals. For example, computer aided detection of tuberculosis by chest x-ray has been used in Zambia, South Africa and The Gambia, and digital ultra sound (using mHealth/telemedicine solutions) has been utilized in Tanzania. Rapid diagnostic tests (RDT) integrated into cloud-based mHealth Smart reader system are in use in Kenya, Tanzania and Ghana, while Smartphone-powered, cloud-enabled portable electrocardiograph (ECG) are used in Uganda and Malawi.

Early adopters of DH on the continent include Malawi, which developed and adopted a national DH (eHealth) strategy in 2003 (2 years before the World Health Assembly action on digital health) (28), Cabo Verde (2007), Ghana (2010), and Kenya (2011) following which several African countries began to adopt DH as a strategy to improve health services delivery (26). Recognizing the need for standardization and comprehensiveness, as well as to furnish countries with standard guidelines to elaborate eHealth strategies, WHO and the International Telecommunication Union (ITU) developed a national eHealth toolkit that articulates three aspects of eHealth strategy development (29). The toolkit sets out the context of the digital health priorities of a country, vision and plan of action for delivering, monitoring, and evaluation of the attainment of the vision. It also articulates the required enabling environment, such as standards, legislation, appropriate technical, and service delivery solutions as well as the required financial and human resources.

As part of its efforts to support regional and national scale up of DH, the African Regional Office of WHO (AFRO) partnered with ITU to strengthen intergovernmental coordination between ministries of health and ICT, and to strengthen private sector engagement, especially with the telecommunication operators. To this effect, both organizations signed a cooperation agreement in October 2017 (30). This agreement has four key areas namely scalability and interoperability, capacity building, stakeholder engagement, and digital clinic innovation. Several other initiatives to strengthen the deployment of DH such as the Digital Regional East African Community Health Initiative (called Digital REACH) (31) and the African Alliance for Digital Health Networks (32) have also been established in the Region.

These DH initiatives were developed against the backdrop that DH is not without its challenges in Africa. Whilst many African countries have developed and adopted DH strategies, implementation of many of such strategies remain slow due to lack of requisite governance framework, institutional capacity, and funding. Most of the pilot projects have not translated into widespread scalable and rational application of DH to health programmes largely due to the fact that the majority of these pilots are project-based, externally funded and unsustainable thus they hardly progress to widespread community use in contrast to in high income countries where DH initiatives are government driven and funded. For example, the Uganda government, in trying to manage the growing number of DH pilots, pronounced a moratorium on DH pilots (33). Uncoordinated piloting of DH initiatives has also resulted in multiple and parallel platforms which are neither interoperable nor integrated, which in turn results in redundancy, and duplication of efforts (34). Lack of a DH capable health workforce is also a challenge which results in low uptake of DH by African health workers (26). Very few African public health learning institutions offer either pre or in-service courses on DH, as a result, there is inadequate awareness and lack of understanding of DH by politicians, decision makers, the older generation of health workers and the ultimate beneficiaries—the community (35). However, the younger generation of African health workers who are more interested in digital technologies are good opportunities for the development of future digital health leaders and workers.

Lack of adequate legal framework and capacity for addressing ethical issues such as digital health data ownership, consent to use, availability and security are also critical hurdles for successful implementation of DH in Africa. Perhaps, the greatest challenges to full realization of the benefits of DH relate to poor fit with existing service delivery approaches, unreliable infrastructure and limited consideration of operational and cultural needs for scalability (10). The skills and expectations for governance and stewardship of DH agenda are still unclear, limiting the health managers' abilities to integrate digital solutions into their work (36). Though the short-term benefits of DH have been demonstrated, proving their longer term public health outcomes and impact has been limited (21). Outside of the domain of the health sector, poor infrastructure such as unstable power supply, unstable and expensive internet connectivity (37), unavailability of maintenance capacities, facilities and advanced technologies which are not adapted to rural African contexts, are critical challenges (21).

## Could DH be a Tool for Health System Strengthening and Achieving UHC?

The foregoing discourse has shown that DH could be used as tools to strengthen health systems for UHC. However, DH is not the panacea to attainment of UHC in Africa. Other prerequisites such as improvement of overall governance and stewardship of health services in a manner that facilitates adoptions of sustainable digital solutions, plus integrating with wider government systems are required. Furthermore, resilient health systems and communities as well as good access to the social, economic and environmental determinants of health are also needed. For instance, the use of drones to deliver blood supplies which has been successfully piloted in Rwanda depended on availability and mobilization of blood donors, kits for blood collection, health workers to carry out the procedures, functional laboratories and blood banks for testing and storage, health workers to administer the blood products to the ultimate beneficiary, liaison with government transport and communication institutions for necessary authorizations and funds to finance the entire system.

Electronic health information systems which have been deployed in several African countries can only achieve their aim if health services are delivered, health information is generated and captured by health workers at the health service delivery points, entered into a database, analyzed and used for informed decision making by public health decision makers. Additionally, the use of rapid SMS systems for community mobilization can only be successful if they transmit health promotion and education products which have been well-designed and produced by health communication experts, are accepted by communities which are well connected digitally, informed and well mobilized; and there are well trained health workers to manage the increased health service uptake generated by such messages. Furthermore, the social, economic and environmental determinants of health such as water, sanitation, good housing etc. which are promoted by rapid SMS messages should be readily available at the community level to support the translation of such messages into improved health outcomes.

Coming back to the central theme of this article; DH could contribute to the sustainable attainment of UHC if there is appropriate DH governance framework, and it is implemented within the broader framework of resilient health systems, communities and access to the social, economic and environmental determinants of health.

## What Needs to be Done to Realize the Potentials of Digital Health in Africa?

Reaping the benefits of DH in Africa requires a number of actions. First, coordinated and synchronized approaches to rapid and wide scale roll out of DH technologies are required. This involves establishment of strong regional and national governance, regulatory and coordination mechanisms, development of appropriate DH national policies, strategies, guidelines and toolkits. This calls for establishment of standards and mechanisms for regional and national integration, adaptability and interoperability of DH technologies (37). Second, development and implementation of legal frameworks that will guide DH data ownership, availability, security, and consent for use are also required. Third, use of context specific DH in the presence of strong health systems, resilient communities and the social determinants of health are also critical for African countries to realize the potential of DH.

Fourth, evaluation of the outcomes, impact and cost-effectiveness, together with sustainable funding models for DH are also required to inform decisions to prioritize DH and determine their feasibility vis-à-vis the context of a country (20, 38). Fifth, bridging the communication, coordination and knowledge gaps between public health and ICT stakeholders is also imperative as well as training a critical mass of human resource for managing and maintaining the DH architecture and infrastructure (39). Sixth, the communities and other stakeholders who are the end-users of DH need to be engaged, mobilized and educated for active participation in DH initiatives. Seventh, Africa should learn and adopt lessons from the successful deployment of DH technologies in other similar settings such as Asia to rapidly advance its quest for DH development. For instance, development and use of offline DH application could help to address internet connectivity challenges in rural African communities (40). Furthermore, adaptation of existing guidance documents from regions with similar contexts could facilitate rapid development of DH strategies which would in turn aid quick deployment of DH technologies (41).

**Figure 1:**
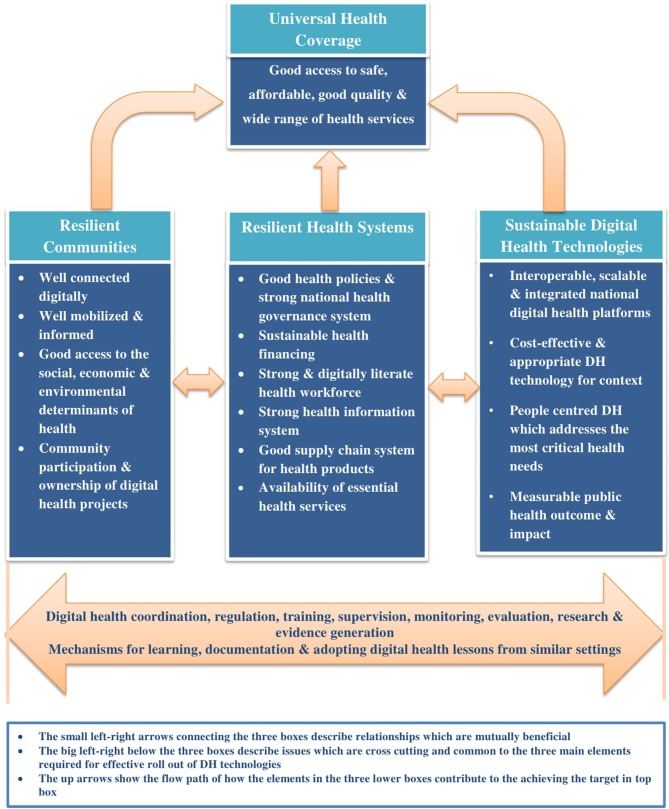
Proposed conceptual framework for rapid and effective scale up of digital health technologies in Africa.

Based on the foregoing, we propose a conceptual framework which is underpinned by resilient communities, strong and functional health systems and sustainable technologies as the basis for effective scale up of DH in Africa (Figure 1). These three main elements should work in tandem to ensure a safe, equitable, efficient access to and coverage of health services.

## Conclusions

Innovative approaches such as DH could leapfrog the attainment of UHC and the other SDG 3 targets in Africa; African health and ICT stakeholders should therefore embrace the transformative capacity which it offers. However, DH initiatives should be implemented within the holistic framework of resilient health systems, communities and the social and economic determinants of health. Although good progress has been made in the deployment of DH in Africa, several challenges militating again its widespread deployment for UHC would need to be addressed. Moving from pilots to concrete and cost-effective DH programmes, which could positively impact the UHC agenda, is an urgent imperative on the continent. In doing so, care should be taken that DH programmes do not divert limited human and financial resources from health services provision and that DH technologies are appropriate for the African context. Given the peculiarities of DH, their deployment requires both intrinsic and extrinsic health systems inputs, high level political commitment, national ownership and appropriate capacity for inter-sectoral coordination, planning, implementation, monitoring, and evaluation.

Further assessments of the opportunities, challenges and landscape for DH in Africa are required to provide evidence-based information for formulation of inspiring DH visions and agenda for change among African political and health leaders. Furthermore, advocacy for stronger DH governance, leadership, funding and development, implementation, supervision, monitoring, and evaluation of foresighted DH strategies and operational plans would provide opportunities to evaluate their outcomes and impacts. The WHO-ITU African DH partnership, African Alliance for Digital Health Networks and Digital REACH initiatives provide platforms for rapidly attaining the above and should be nurtured to fruition by all concerned stakeholders.

## Author Contributions

OO conceived, coordinated the study and wrote the first draft of the manuscript. DM participated in the conception of the study, writing of the first draft of the manuscript and overall coordination of the study. JB, M-RN, HB, YT, HK, and DD contributed to the writing of all subsequent drafts of the manuscript and review of literature. All authors read and provided significant inputs into all the drafts of the manuscript, agreed to be accountable for all aspects of the work and approved the final draft of the manuscript for publication.

### Conflict of Interest

The authors declare that the research was conducted in the absence of any commercial or financial relationships that could be construed as a potential conflict of interest.

## Footnotes

^1^In the context of this article, Africa refers to the 47 Members States of AFRO majority of which are in sub-Saharan Africa: Algeria, Angola, Benin, Botswana, Burkina Faso, Burundi, Cabo Verde, Cameroon, Central Africa Republic, Chad, Comoros, Cote d'Ivoire, Democratic Republic of the Congo, Equatorial Guinea, Eritrea, Ethiopia, Gabon, the Gambia, Ghana, Guinea, Guinea Bissau, Kenya, Lesotho, Liberia, Madagascar, Malawi, Mali, Mauritania, Mauritius, Mozambique, Namibia, Niger, Nigeria, Republic of Congo, Republic of South Sudan, Rwanda, Sao Tome and Principe, Senegal, Seychelles, Sierra Leone, South Africa, Swaziland, Togo, Uganda, United Republic of Tanzania, Zambia and Zimbabwe.

## Abbreviations

AFRO: African Regional Office of the World Health Organization
DH: digital health
ICT: information and communication technology
ITU: International Telecommunication Union
SDGs: sustainable development goals
UHC: Universal Health Coverage
WHO: World Health Organization.
